# Hypertensive disorders during pregnancy and elevated blood pressure in the offspring: A systematical review and meta-analysis protocol

**DOI:** 10.1097/MD.0000000000015677

**Published:** 2019-05-17

**Authors:** Huan Yu, Yuan He, Zhengxia Mao, Wenbing Dong, Xiaodong Fu, Xiaoping Lei

**Affiliations:** a Department of Neonatology; b Department of Gynaecology and Obstetrics, Affiliated Hospital of Southwest Medical University, Luzhou, Sichuan, China.

**Keywords:** blood pressure, hypertensive disorders during pregnancy, meta-analysis, preeclampsia

## Abstract

**Background:**

Hypertensive disorders during pregnancy (HDP), including gestational hypertension, preeclampsia (PE), and chronic hypertension, affect approximately 10% of pregnancies worldwide. PE was associated with elevated blood pressure (BP) in the offspring confirmed by previous literature. No previous systematic review has investigated the associations between the subtypes of HDP and BP in offspring. Therefore, from the published literature, the present systematic review and meta-analysis aims to reach to a clear consensus on this topic.

**Methods:**

The Cochrane Handbook for Systematic Reviews of Interventions was used to structure our methodological approach, and the Preferred Reporting Items for Systematic Reviews and Meta Analyses Protocols guidelines to this protocol. We will include cohort, case-control, and cross-sectional studies, in which HDP or its subtypes were reported and BP in the offspring was the outcome of interest. A systematic search of PubMed, Embase, the Cochrane library, and Web of Science will be conducted according to a specific search strategy. The titles and abstracts of all literature will be reviewed independently by 2 authors. A standardized data collection form will be used for data extraction. The overall pooled estimates will be calculated by meta-analysis in Revman 5.3 based on an inverse variance method. According to the heterogeneity by the *I*^2^, a random-effect model or a fixed-effect model will be chosen to pool data. Risk of bias and quality of the studies will be assessed by a suitable bias classification tool.

**Results:**

This study will provide high-quality evidence of the effects in offspring BP, which were influenced by different subtypes of HDP.

**Conclusion:**

This systematic review will infer a conclusion on the associations between different subtypes of HDP and BP in offspring.

**Ethics and dissemination:**

Due to the data come from the published study, the present systematic review and meta-analysis does not require ethics approval. Findings will be published in a peer-reviewed journal.

**Registration:**

Supporting data can be found at the International Prospective Register of Systematic Reviews (PROSPERO) website, with registration number CRD 42018110872.

## Abstract 2

Supplemental Digital Content is available in the text

## 1 Introduction

The “Developmental Origins of Health and Disease (DOHaD)” hypothesis has been widely proved by epidemiological studies.^[1]^ The fetuses, exposure to the poor uterine environment, have high risks of chronic noncommunicable diseases in later life, including hypertension, diabetes, and obesity.^[2]^

Hypertension, is one of the most serious public health burdens and has sharply increasing worldwide.^[3,4]^ Hypertension-related mortality and morbidity is the largest contributor to global disability-adjusted life-years.^[3–5]^ Although hypertension is a complex multifactor disorder, an increasing number of studies implied hypertension have a programmed or developmental origin,^[6,7]^ meaning that the adverse intrauterine environment may be an important independent risk factor for blood pressure (BP) in later life.

Hypertensive disorders during pregnancy (HDP) is one of the most common hostile utero environments of various pathophysiological changes, including systemic inflammation and oxidative stress, affecting approximately 10% of pregnancies worldwide.^[8,9]^ HDP include any hypertensive conditions before gestation or with manifestation before 20 weeks, and hypertension starting at or after 20 weeks.^[9,10]^ It mainly includes gestational hypertension (GH), preeclampsia (PE), chronic hypertension, and PE superimposed on chronic hypertension.

There is a younger trend of hypertension and the prevalence rate of childhood hypertension runs up to 3.3%.^[11,12]^ A BP trajectory was observed in life from childhood to adulthood, and indicated that higher BP in childhood was prone to develop hypertension in adult.^[13,14]^ Previous studies also showed that HDP could alter the development of fetal vasculature and cardiac structure,^[15,16]^ and induce higher BP of the offspring in later life.^[15]^ Thus, exposure to HDP during critical periods of development may predispose offspring to develop elevated BP later in life, and it may be a critical period for early intervention of hypertension.^[17]^

## 2 Rationale for current systematic review

Several reviews had summarized the cardiometabolic adverse effects in the offspring of mothers suffer from PE.^[18–20]^ Two previous meta-analyses in 2009 and 2012 higher systolic and diastolic pressure in children were associated with exposure to PE in the utero.^[21,22]^ GH, absence of proteinuria, was also associated with higher BP in the offspring.^[23,24]^ However, some new studies were conducted on this topic in the past several years, and inconsistent associations were observed between the subtypes of HDP and the offspring's BP.^[13,25–28]^ In one of these studies, GH, PE, and chronic hypertension were associated with elevated systolic BP in the offspring, but no such associations with diastolic BP.^[26]^ And in another one,^[13]^ the BP in the offspring was not associated with PE, although the positive associations existed in GH and chronic hypertension. Furthermore, in some studies, no elevated BP was observed in the offspring born in mothers with any types of HDP.^[29]^ In addition, the cardiovascular effects of offspring after intrauterine exposure to HDP were demonstrated by 2 reviews recently.^[30,31]^ Unfortunately, not all studies were included and HDP was not categorized to different types for further analysis. So far, no clear associations have already disclosed between different subtypes of HDP and the offspring's BP, and a meta-analysis need to be conducted to deep analyze these associations based on the published literature.

## 3 Objective

The aim of the present systematic review and meta-analysis, based on the current studies, is to summarize the available evidences and examine the associations between different subtypes of HDP and the offspring's BP in exposed children.

## 4 Method and design

The Cochrane Handbook for Systematic Reviews of Interventions was used to structure our methodological approach, and the Preferred Reporting Items for Systematic Reviews and Meta Analyses (PRISM-A) Protocols guidelines to this protocol.^[32]^ We will report our findings following PRISM-A guidelines.

## 5 Eligibility criteria

### 5.1 Population

Pregnant women and their offspring exposed in utero to HDP, GH, or PE.

### 5.2 Intervention (exposure)

According to the American College of Obstetricians and Gynecologists and the International Society for the Study of Hypertension in Pregnancy criteria,^[9,10]^ HDP is any hypertensive disorders during pregnancy, mainly including GH, PE, chronic hypertension, and PE superimposed on chronic hypertension. Hypertension is defined as BP ≥ 140/90 mmHg or a rise of 30 or 15 mmHg from baseline level.

HDP: any hypertensive disorders during pregnancy.

GH: new-onset hypertension alone after gestational 20 weeks without proteinuria.

PE: new-onset hypertension accompanied by proteinuria or together with other evidences of systemic involvements (include renal insufficiency, elevated levels of liver transaminases, pulmonary oedema, thrombocytopenia, et al), after gestational 20 weeks.

Pregnancy-associated hypertension (PAH): hypertension occurring after gestational 20 weeks, including PE and GH.

Chronic hypertension with pregnancy: hypertension before 20 weeks of gestation or persisting beyond 12 weeks postpartum.

### 5.3 Comparison

No diagnosis of HDP (including PAH, PE, and GH).

### 5.4 Outcomes

1. Primary outcome: mean difference in offspring's BP, whose mothers with and without different types of HDP.
2. Secondary outcome: mean BP in offspring of mothers with different types of HDP.

### 5.5 Inclusion criteria

1. We will include case-control, cohort, and cross-sectional studies, which reported mean difference in BP or mean BP of the offspring of mothers with or without different types of HDP.
2. We collect the data, coming from an original study, in which different types of HDP were confirmed by diagnostics from standard medical institutions, including medical records, and diagnostics by the obstetrician.
3. We will include peer-reviewed literature published in English as possible, during the period from the time of data inception to Nov. 2018.

### 5.6 Exclusion criteria

1. Studies published are not written in English.
2. We will exclude case reports, case series, active reviews, expert opinion, letters, and the articles only with abstract in conference paper.

## 6 Search strategy

The reviewers conduct a systematic literature search in the following electronic databases: PubMed, Embase, Web of Science and the Cochrane library. Comprehensive electronic literature search strategies, according to the principles of Boolean Logic, will be used for each database (see Text, Supplement content, which demonstrates all search terms and a detail research strategy in PubMed). We also intend to check reference lists from the included studies for any important studies missed during the database search.

## 7 Data collection

To select studies for further assessment, the Endnote manager will be used to store and manage titles and abstracts of all the studies from each database. Two independent reviewers will screen all the titles and abstracts by using the predetermined inclusion and exclusion criteria. Records identified as potentially eligible on the basis of title and abstract, full texts will be obtained to screen. While an eligible study does not provide full data, we will contact the corresponding authors to obtain data if possible. Where consensus on eligibility cannot be achieved, a third review author will be involved in the discussion. Two independent authors undergo data extraction using a standardized data collection form (see Tables, Supplement content, which demonstrates the detail information of extraction data), from the included studies, and use the PRISM-A flow diagram to demonstrate the included and excluded studies and reasons in the end. When the same cohort was reported in multiple articles, the study which contains the largest sample is included as possible.

The original standardized data collection form was drafted by several authors, basing data extraction form on the data collection form from the Cochrane. In the data collection form, data extraction mainly includes general information of study, characteristics of included studies, the information and outcome of subgroup, risk of bias assessment and data analysis. General information of study includes first author, date of publication, country of publication, participants, type of study, and type of outcome measurements. Characteristics of included studies mainly include sample size, inclusion and exclusion criteria, number of cases and controls, definition of HDP, PE, and GH, etc. The subgroup information Data analysis includes confounders adjusted, such as age, gender, the time of follow up, the main results, et al. If required, a third reviewer will be consulted.

## 8 Appraisal of quality and risk bias

Quality assessment will be conducted by 2 reviewers independently basing on an appropriate assessment tool according to the study design. If any disagreement, a third reviewer will discuss it with the 2 reviewers. The Newcastle-Ottawa Scale (NOS) is used to evaluate the risk bias of the included studies.^[33]^ The NOS assessment consisted of 3 major categories: selection (1 star for each terms), comparability (up to 2 stars), and exposure (1 star for each terms). Study with high score, has lower risk of bias. The scale of the Agency for Healthcare Research and Quality (AHRQ) for cross-sectional studies. For appraising an overall likelihood of quality, the Grading of Recommendations Assessment, Development and Evaluation (GRADE) may was used to assess 6 aspects of every study (selection, exposure, outcome, analytic, attrition, and confounding). The quality of studies will be classified as minimal, low, moderate, and high, and the overall likelihood of quality based on the total of the 6 types will be measured.

## 9 Data analysis

The mean differences in BP of offspring born in mothers with and without HDP will be used to estimate effect sizes by using RevMan 5.3. In the next step, PAH, GH, PE, and chronic hypertension will be chose to conduct the same analysis, respectively. We will use the generic inverse variance method to display the results. If possible, the analysis will be performed in adjusted results to assess the changes in the relationship after adjusting confounders. If we are unable to analyze data using meta-analysis, a narrative synthesis will be conducted to summarize and tabulate the results.

## 10 Assessment of heterogeneity

We will assess heterogeneity by visual assessment of forest plots and the statistic of *I*^2^ value. While heterogeneity is low (*I*^2^ < 50%), a fixed-effects model will be used, and while heterogeneity high (*I*^2^ ≥ 50%) a random-effects model used according to the Cochrane Handbook criteria.^[34]^ The following subgroup and sensitivity analyses using RevMan 5.3 will be performed, where the data allow:

1. according to study design (case-control vs cohort);
2. according to study quality (minimal/low vs moderate/high);
3. according to age of the HDP offspring.

We will conduct funnel plots to assess the potential for publication bias where there are sufficient (>10) studies. If there are subgroup and sensitivity analyses in the process of the meta-analysis, we will conduct such some post hoc analyses to explore potential high heterogeneity or publication bias.

## 11 Reporting of results

The study selection process will be represented in a flow diagram (as stated in the PRISM-A statement), providing reasons for excluding research step by step. Two tables will list the characteristics and quality assessment of the included studies. Publication bias will be presented using forest plots. The eligible studies we could not obtain raw data by contacting corresponding authors, will be listed individually in a separate table. If a meta-analysis unable to be conducted, a narrative synthesis will be done.

## 12 Conclusion

This systematic review and meta-analysis will summarize eligible studies to examine the associations between different subtypes of HDP and BP of offspring according to the prepared protocol, which may help to identify the possible contributors to hypertension. Therefore, by examining the mean difference in BP, it may demonstrate that different subtypes of HDP have different effects on BP of offspring. It may help to be aware of some HDP needing more active reasonable intervention.

## 13 Potential limitations

There are some several potential limitations in our study. First, the articles with positive results are more likely to being published and we only search the articles published in English, leading to publication bias. We will use a funnel plot to assess the publication bias. Second, due to definitions of HDP, PE, and GH varied during different periods, we could not exclude the possibility that studies could not meet current criteria, and we will ensure the stability of results by exploring subgroups. Third, the potential confounders are also a potential limitation. The same analysis will display both on the crude and adjusted results, possibly helping to attenuate these limitations. There are some potential confounders (such as the treatment of HDP, other maternal mental illness, etc) not clear, and different confounders adjusted in different studies (e.g., maternal pregnancy body mass index, maternal smoking), but our study cannot provide insight into mechanisms underlying this phenomenon. For example, previous studies demonstrated that birth weight and preterm birth area strong mediate factors between HDP and BP in offspring.^[35,36]^ And raised maternal pregnancy body mass index is associated with both PE and higher offspring's BP.^[37]^ Conversely, some factor maternal smoking increases offspring BP in adult life,^[38]^ although it reduces the risk of PE by up to 50%.^[39]^

## Author contributions

HY is the guarantor of the article. The manuscript was drafted by HY. HY and XL developed the search strategy. HY, YH and XM independently screened the potential studies and extracted data and planned quality appraisal of included studies. XF and XL arbitrated any disagreement and ensure that no errors occur during the review. All review authors critically reviewed, revised, and approved the subsequent and final version of the protocol.

**Conceptualization:** Huan Yu, Xiaodong Fu, Xiaoping Lei.

**Data curation:** Huan Yu, Yuan He, Zhengxia Mao.

**Formal analysis:** Huan Yu.

**Funding acquisition:** Xiaoping Lei.

**Methodology:** Huan Yu, Xiaoping Lei.

**Writing – original draft:** Huan Yu.

**Writing – review & editing:** Yuan He, Zhengxia Mao, Wendin Dong, Xiaodong Fu, Xiaoping Lei.

## Supplementary Material

Supplemental Digital Content
