# Supplementary material for: Hypertensive disorders during pregnancy and elevated blood pressure in the offspring: A systematical review and meta-analysis protocol
Source: Medicine (Baltimore). 2019 May 17;98(20):e15677. doi: 10.1097/MD.0000000000015677 (PMC6531162; doi:10.1097/MD.0000000000015677)
Supplement: Supplemental Digital Content [file medi-98-e15677-s001.pdf]

Title：Hypertensive Disorders During Pregnancy and Elevated Blood Pressure in the Offspring: a Systematical Review and Meta-analysis Protocol

First author: Huan Yu

Search term in all electronic databases

1. pregnancy-induced hypertension
2. pregnancy induced hypertension
3. pregnancy transient hypertension
4. hypertensive disorders of pregnancy
5. hypertensive pregnancy disorder
6. hypertensive disorders in pregnancy
7. hypertensive disorders complicating pregnancy
8. pregnancy hypertension
9. gestational hypertension
10. gravidic hypertension
11. gravid hypertension
12. pre eclampsia
13. preeclampsia
14. eclampsia

15. offspring
16. newborn
17. children
18. kids
19. adolescents
20. infants
21. neonatal
22. young people
23. teenagers
24. younger generations

25. blood pressure
26. hypertension

27. [#1 or #2 or #3 or #4 or #5 or #6 or #7 or #8 or #9 or #10 or #11 or #12 #13 or #14]
28. [#15 or #16 or #17 or #18 or #19 or #20 or #21 or #22 #23 or #24]
29. [#25 or #26]

Final search strategy: [#27 and #28 and #29] Filters: Humans; English

This is the search strategy in PubMed:

Search ((((((((((((offspring[Title/Abstract]) OR newborn[Title/Abstract]) OR children[Title/Abstract]) OR kids[Title/Abstract]) OR adolescents[Title/Abstract]) OR infants[Title/Abstract]) OR neonatal[Title/Abstract]) OR young people[Title/Abstract]) OR teenagers[Title/Abstract]) OR younger generations[Title/Abstract]))) AND (((blood pressure[Title/Abstract]) OR hypertension[Title/Abstract]))) AND (((("Pre-Eclampsia"[Mesh]) OR "Hypertension, Pregnancy-Induced"[Mesh]) OR (((((((((((((pregnancy-induced hypertension[Title/Abstract]) OR pregnancy induced hypertension[Title/Abstract]) OR gestational hypertension[Title/Abstract]) OR pregnancy transient hypertension[Title/Abstract]) OR hypertensive pregnancy disorder[Title/Abstract]) OR hypertensive disorders of pregnancy[Title/Abstract]) OR hypertensive disorders in pregnancy[Title/Abstract]) OR hypertensive disorders complicating pregnancy[Title/Abstract]) OR pregnancy hypertension[Title/Abstract]) OR gravidic hypertension[Title/Abstract]) OR gravid hypertension[Title/Abstract]) OR pre eclampsia[Title/Abstract]) OR preeclampsia[Title/Abstract]) OR eclampsia[Title/Abstract]))) Filters: Humans; English

Title：Hypertensive Disorders During Pregnancy and Elevated Blood Pressure in the Offspring: a Systematical Review and Meta-analysis Protocol

First author: Huan Yu

Data collection form

Date of data collection	
Review title	
Study authors	
Notes	

Study details

First author	
Contact details	
Year of publication	
Country of publication	
Notes	

Study eligibility

Study Characteristics	Eligibility criteria	Eligibility criteria met?<br>Yes No Unclear	Location in text
Type of study<br>(case-control, cohort)			
Participants			
Types of intervention			
Types of outcome measures			
Include ☐		Exclude ☐	
Reason for exclusion			
Notes:			

DO NOT PROCEED IF STUDY EXCLUDED FROM REVIEW

Characteristics of included studies

	Descriptions		Location
Population description (area/country, hospital, study queue name, year)			
Total population			
Finally population			
Race			
Follow-up, year (mean)			
Age of pregnancy			
Pre-pregnancy BMI			
Gestational hypertension			
chronic HBP			
Preeclampsia			
child Gender（m/fe）			
Inclusion criteria			
Exclusion criteria			
Measuring method			
Definition of HDP			
Definition of PE			
Definition of GH			
Definition of chronic HBP			
Definition of other HBP			
Definition of hypertension			
Ethical approval	Yes No Unclear		
Informed consent	Yes No Unclear		
Notes:			

Subgroup

Group:

	Descriptions	Location
**Material**		
The total number		
Material age, year		
Material BMI, kg		
Maternal BP, mmHg		
Educational level		
Smoking during pregnancy		
alcohol		
Material diabetes		
**offspring**		
Birth weight, g		
Gestational age, w		
Offspring gender (male /female)		
Age		
Weight, kg		
Height, cm		
BMI, kg m^{-2}		
Notes:		

Outcome

Group:

		Description	Location
BP	SBP		
	DBP		
BP strict criteria	SBP		
	DBP		
mean difference	Model		
	SBP		
	DBP		
OR 95% CI	SBP		
	DBP		
regression coefficients (β，95%CI)			
	SBP		
	DBP		
Notes:			

Risk of Bias assessment

For cohort studies

Domain	Item	Score
Selection	Representativeness of the exposed cohort	a) truly representative of the average ___ (describe) in the community<br>b) somewhat representative of the average____ in the community<br>c) selected group of users eg nurses, volunteers<br>d) no description of the derivation of the cohort
	Representativeness of the non-exposed cohort	a) drawn from the same community as the exposed cohort<br>b) drawn from a different source<br>c) no description of the derivation of the non exposed cohort
	Ascertainment of exposure	a) secure record (eg surgical records)<br>b) structured interview<br>c) written self report d) no description
	Demonstration the outcome of interest was not present at start of study	a) yes<br>b) no
Comparability	Comparability of cohort on the basis of the design or analysis	a) study controls for ___ (select the most important factor)<br>b) study controls for any additional factor (This criteria could be modified to indicate specific control for a second important factor.)
Outcome	Assessment of outcome	a) independent blind assessment<br>b) record linkage<br>c) self report<br>d) no description
	Was follow-up long enough for outcomes to occur	a) yes (select an adequate follow up period for outcome of interest)<br>b) no
	Adequacy of follow up of cohort	a) complete follow up - all subjects accounted for<br>b) subjects lost to follow up unlikely to introduce bias - small number lost - > ___ % (select an adequate %) follow up, or description provided of those lost)<br>c) follow up rate < ___% (select an adequate %) and no description of those lost d) no statement
Quality scores		

For case control studies

Domain	Item	Score
Selection	Is the case definition adequate	a) yes, with independent validation<br>b) yes, eg record linkage or based on self reports<br>c) no description
	Representativeness of the cases	a) consecutive or obviously representative series of cases<br>b) potential for selection biases or not stated
	Selection of Controls	a) community controls<br>b) hospital controls<br>c) no description
	Definition of Controls	a) no history of disease (endpoint)<br>b) no description of source
Comparability	Comparability of cases and controls on the basis of the design or analysis	a) study controls for ____ (Select the most important factor.)<br>b) study controls for any additional factor (This criteria could be modified to indicate specific control for a second important factor.)
Exposure	Ascertainment of exposure	a) secure record (eg surgical records)<br>b) structured interview where blind to case/control status<br>c) interview not blinded to case/control status<br>d) written self report or medical record only<br>e) no description
	Same method of ascertainment for cases and controls	a) yes<br>b) no
	Non-Response rate	a) same rate for both groups<br>b) non respondents described c) rate different and no designation
Quality scores		

Data analysis

	Description as stated in report/paper									Location
Comparison										
Outcome										
Subgroup										
Time point										
Results										
	mean	SD	N	mean	SD	N	mean	SD	N	
SBP										
DBP										
Any other results reported (e.g. mean difference, CI, P value)										
Statistical methods used and appropriateness of these (e.g. adjustment for correlation)										
Reanalysis required? (specify, e.g. correlation adjustment)	Yes No Unclear			-						
Reanalysis possible?	Yes No Unclear			-						
Reanalysed results	-									
Notes:										

Other information

	Description as stated in report/paper	Location
Key conclusions		
Study funding sources		
conflicts of interest		
References to other relevant studies		
Correspondence required for further study information *(from whom, what and when)*		
Notes:		
